# Serum concentration of calcium, magnesium and zinc in normotensive versus preeclampsia pregnant women: A descriptive study in women of Kerman province of Iran

**Published:** 2015-01

**Authors:** Homeira Vafaei, Maryam Dalili, Seyed Amin Hashemi

**Affiliations:** 1 *Maternal-fetal Medicine Research Center, Shiraz University of Medical Sciences, Shiraz, Iran.*; 2 *Clinical Research Unit, Afzalipour Hospital, Kerman University of Medical Sciences, Kerman, Iran.*

**Keywords:** *Preeclampsia*, *Pregnancy*, *Calcium*, *Magnesium*, *Zinc*

## Abstract

**Background:**

Preeclampsia is a disorder of pregnancy without any specific reasons that characterized by high blood pressure and large amounts of protein in the urine. This disorder is caused by multiple factors and finding any factor related to this disorder can help on time prevention of this disease.

**Objective:**

In this study, serum levels of calcium (Ca), magnesium (Mg) and zinc (Zn) were evaluated in preeclampsia women and compared to normotensive ones.

**Materials and Methods:**

This was a case-control study on 40 normotensive pregnancies as controls, 20 mild and 20 severe preeclamptic pregnancies as case groups. The women were studied in their 28-40 weeks of pregnancy. Simple random sampling was done based on inclusion and exclusion criteria and data were collected by blood sampling.

**Results:**

The serum Ca levels of 4.96±0.62, 4.89±0.34, 5.05±0.35 mg/dL, Mg levels of 0.83±0.08, 0.85±0.11, 0.84±0.11 mg/dL and Zn levels of 107.55±22.74, 108.00±22.40, 107.50±22.30 mg/dL was detected in normotensive, mild and severe preeclampsia, respectively. Statistical analysis revealed that there were no significant differences between three groups in serum levels of Ca (p=0.6), Mg (p=0.827) and Zn (p=0.997).

**Conclusion:**

The findings of this study showed that the assessment of serum Ca, Mg and Zn levels does not have any clinical values for predicting and/or managing of preeclampsia. However, based on the positive relationship between serum Ca and Mg concentration and the severity of preeclampsia in this study, we recommend assessment of serum levels of these two mineral elements as indices of the severity of preeclampsia.

## Introduction

Although, pregnancy is a normal physiological state in the maternal environment, but itself and its complications are the cause of about 600,000 women death, every year in the word and half of them are due to risky pregnancies (1). Preeclampsia (PE), as a risky pregnancy, is a systemic disease characterized by hypertension, proteinuria and edema, which are thought to be the result of diffuse endothelial activation and dysfunction (2). About 5% of all pregnant women have PE during the second half of gestation that can cause maternal death throughout the world and is accompanied by substantial perinatal morbidity and mortality (3). PE is the third common cause of maternal death in the world and the second common cause of mothers mortality in Iran, 18% of maternal death in Iran is due to PE (4). The search for finding the causative factors of this disorder has therefore been a major focus of obstetrical investigation and in spite of several-decade studies on PE, the causes are still unknown. Imbalance between lipid peroxidation and antioxidants status in PE has been suggested as factors contribute to the damage of endothelium (2, 5). Minerals have important influence on the health of pregnant women and growing fetus. Among them, serum or placental zinc (Zn) concentrations have been reported to be low or unchanged in PE women (6, 7). 

Magnesium (Mg), the second most abundant intracellular cation after potassium has been identified as a cofactor in over 300 enzymatic reactions involving energy metabolism, protein and nucleic acid synthesis (8). Also, disturbance in metabolism of essential micronutrients like calcium (Ca) and Mg may play an important role in the development of PE (9). Inadequate intake of Ca plays a contributory role in the pathogenesis of hypertension and the hypomagnesaemia increases the risk of pregnancy complications. (10). On the basis of the above mentioned findings, the present study was designed to examine changes in levels of serum Cu, Mg and Zn to assess the possible relationship between these parameters in the pathogenesis of PE.

## Materials and methods

**Patients**

At first the study was approved by Ethics Committee of Kerman University of Medical Sciences and written consent was obtained from all participants. 80 pregnant women were included in this case-control study, which were selected from Obstetrics and Gynecology Department, Afzalipour Hospital, Kerman University of Medical Sciences, Kerman, Iran between March to August 2012. 

**Selection criteria**

Our inclusion criteria were singleton pregnancy with alive baby, gestational age of 28-40 weeks, and BMI of 17-29.9 Kg/m^2^ at reception time. Participant with one of the following parameters were excluded from our study: fetal abnormalities, underlying diseases, infections, immunological disorders, alcohol or drug abuse, smoking, history of infertility, obstetric complications such as placental abruption or previa, consumption of anti-cancer, immunosuppressive and anticoagulant drugs, aspirin, calcium or other medications that contain zinc. Blood pressure (BP) more than 140/90 and proteinuria >300 mg/dL in 24 hr urine or 1+ in dipstick urine sample were the basic criteria for diagnosis of PE. These two criteria alone is used for mild PE patient selection and severe PE was considered if one of the following points was include; BP more than 160/110, proteinuria >5000 mg/dL in 24 hr urine or 3+ in dipstick two consecutive urine samples within 4 hr, oliguria with urination volume <500 ml/d, microangiopatic hemolysis, thrombocytopenia, epigastric pain with elevation in serum hepatic enzyme, persistent neurologic symptoms or blindness (petechiae or occipital bleeding), headache and blurred vision, oligohydroamniuos and fetal IGUR. History of fetal defects, medication or any other disease must not be seen in all subjects. Normotensive subjects had all fulfills previously mention criteria but do not develop hypertension during whole pregnancy. Subjects were divided into three groups. Forty women represented as normotensive group, and other subjects were divided equally between mild preeclampsia (MPE) and severe preeclampsia (SPE) groups.

**BP and proteinuria measurements**

BP of all subjects was measured by the sphygmomanometer in two times with 6 hr distance. For diagnosing proteinuria, the protein content of 24 hr urine sample was measured routinely or 2 midstream urine samples collected in morning and evening showing albumin ”±” in reagent stripe.

**Estimation of serum Ca, Mg, and Zn**

Serum Ca was estimated by o-Cresolphthalein complex one, without deproteinization method. Calcium forms a violet complex with o-Cresolphthalein complex one in an alkaline medium. Serum Mg was estimated by xylidyle blue. Magnesium ions reacts with xylidyl blue in an alkaline medium to form a water soluble purple red chelate, the color intensity of which is proportional to the concentration of magnesium ion in the sample. Calcium is excluded from the reaction by complexing with EGTA. Serum Zn concentration was assayed by routine laboratory method using autoanalyzer.

**Statistical analysis**

All data concerning age and serum levels of Ca, Mg, and Zn were expressed as the mean±SD and analyzed by Kruskal-Wallis test using the SPSS statistical package (SPSS 17, SPSS Ltd., Working, Surrey, UK). The Tukey’s test was carried out to assess any significant differences. P<0.05 was accepted as being statistically significant.

## Results

**Figure 1:**
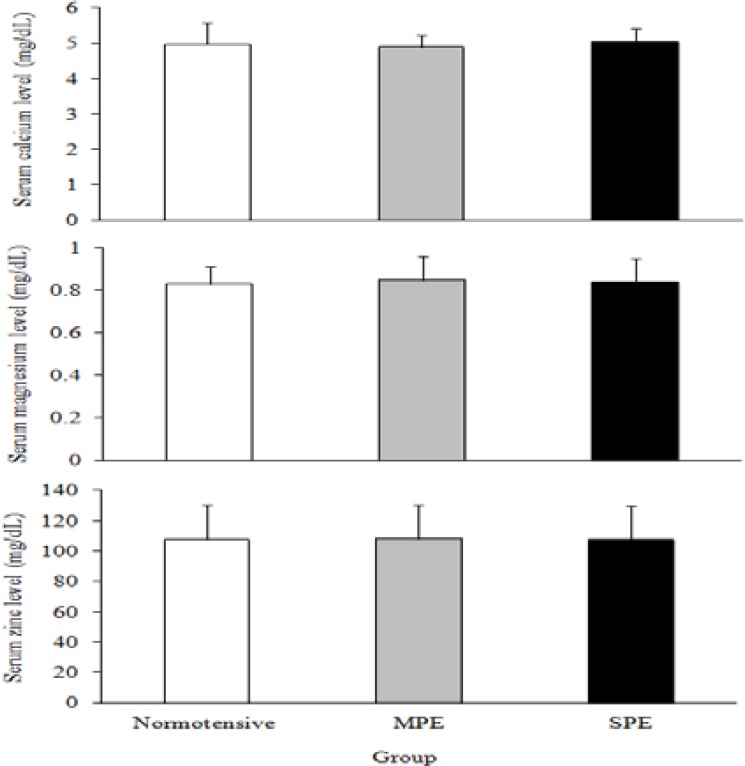
Mean±SD of serum concentration of calcium, magnesium and zinc in normotensive (n=40), mild preeclampsia )MPE( (n=20) and severe preeclampsia )SPE( (n=20). The Tukey’s test was carried out to assess any significant differences. There were no significant differences between normotensive, mild and severe preeclampsia in serum levels of calcium, magnesium or zinc (p>0.05).

Total and group based descriptive analysis of mothers age is presented in table I. These data demonstrated that mothers in age group of 28-33 years had the most frequency of MPE and SPE. In normotensive peoples, the most frequency of participants was detected in age group of 22-27 years. Serum levels of Ca, Mg, and Zn in normotensive, MPE and SPE groups are presented in figure 1. These data showed that there were no significant differences in none of three elements between normotensive subjects with MPE, and SPE, and also between MPE and SPE patients (p=0.62, p=0.82 and p=0.99, respectively).

**Table I:** Descriptive analysis of mother’s age in different groups

**Age group (years)**	**Normotensive**		**MPE**		**SPE**		**Total**	
	**Frequency**	**%**	**Frequency**	**%**	**Frequency**	**%**	**Frequency**	**%**
15-21	7	17.5	3	15	3	15	13	16.3
22-27	14	35	5	25	5	25	24	30
28-33	13	32.5	7	35	8	40	28	35
34-39	6	15	5	25	4	20	15	18.8
Total	40	100	20	100	20	100	80	100

MPE: mild preeclampsia

SPE: severe preeclampsia.

## Discussion

Concentrations of various trace elements are altered during pregnancy with changes in the mother’s physiology and the requirements of growing fetus. Changes on levels of Cu, Ca, and Mg during all the three durations of pregnancy and Zn during mid and late pregnancy and postpartum were reported previously (11). In addition, it has been reported that reduction in serum levels of Ca, Mg, and Zn during pregnancy might be possible contributors in etiology of PE, and supplementation of these elements to diet may be of value to prevent PE (12). 

In the present study we found that serum Ca, Mg, and Zn levels in PE pregnant women had no significant differences with normotensive subjects and also severity of this disorder could not influenced the serum levels of these metal elements. Ca and Mg are very important micronutrients and involves in various cellular mechanisms like muscle contractility. Blood vessels require sufficient amount of Ca to contract and Mg to relax and open up to regulate the normal BP (9). Also, the recent literature indicates that alterations in plasma and cellular zinc are associated with various obstetric pathologies (13). Based on a cross-sectional study conducted in 2011, it had been concludes that serum Ca and Zn deficiency may be one of the risk factor of PE (14). Malas and Shurideh reported that during pregnancy due to the increase in glomerular filtration rate, calciuria increases along with removal of more Ca by transfering it to the fetus so maternal calcium levels come down (15). 

Although, in our study, the serum levels of Ca was lower and higher in MPE and SPE than these levels in normotensive subjects, respectively, but these changes were not significant. Das and his colleagues reported that serum Mg level of PE pregnant women was significantly higher compared to normotensive pregnant women (8). Also Harma and his collaborators reported the higher concentration of Ca and Zn in serum of PE women compare to normotensive ones (16). In another study, it was reported that the serum Zn level in PE subjects was insignificantly lower than that in normal pregnancy group, which is in agreement with our results (1). 

Our finding about Zn concentration in the serum of PE patients also is supported by the results of some other studies. In Kanagal *et al* study the serum Ca concentration was significantly lower in the PE group compared to normotensives, whereas the levels of serum Mg showed a marginal difference in both groups (17). In another study directed by Adam *et al* also the mean serum Zn in PE was lower than that in normal pregnancy (18). Controversial to the results of the present study, in a study by Ilhan *et al* the mean serum Zn level in normal pregnancy group was lower than PE and the difference was statistically significant (6). The disagreements which were seen between our results and some of other previously reports may be due to difference in age of mothers, nutrition, pregnancy age and some other latent reasons. 

In conclusion, there were no limitations but dissatisfaction of some patients in our study. Although it is concluded that serum Ca, Mg and Zn can be considered as factors having a role in the etiopathogenesis of the disease and as severity indicators in preeclamptic women, but we found no differences in serum levels of these three elements between normotensive and PE subjects and no relationship between the serum levels of them with severity of PE. However, nutritional health education should be used as a preventive approach to allow the large sector of the developing countries population to maximize the use of the limited resources in the best way.

## Note

***This article extracted from M.D. thesis. (Seyed Amin Hashemi)***
